# mGlu5 receptor availability in youth at risk for addictions: effects of vulnerability traits and cannabis use

**DOI:** 10.1038/s41386-020-0708-x

**Published:** 2020-05-15

**Authors:** Sylvia M. L. Cox, Maria Tippler, Natalia Jaworska, Kelly Smart, Natalie Castellanos-Ryan, France Durand, Dominique Allard, Chawki Benkelfat, Sophie Parent, Alain Dagher, Frank Vitaro, Michel Boivin, Robert O. Pihl, Sylvana Côté, Richard E. Tremblay, Jean R. Séguin, Marco Leyton

**Affiliations:** 1 Department of Psychiatry, McGill University, Montreal, QC Canada; 2 Department of Neurology & Neurosurgery, Montreal Neurological Institute, McGill University, Montreal, QC Canada; 3 Department of Cellular and Molecular Medicine, University of Ottawa, Ottawa, ON Canada; 4 Institute of Mental Health Research, Affiliated with the University of Ottawa, Ottawa, ON Canada; 5 Yale PET Center, Yale University, New Haven, CT USA; 6 School of Psychoeducation, Université de Montréal, Montreal, QC Canada; 7 CHU Ste-Justine Research Center, Montreal, QC Canada; 8 Department of Psychology, Université Laval, Quebec, QC Canada; 9 Department of Psychology, McGill University, Montreal, QC Canada; 10 Department of Social and Preventative Medicine, Université de Montréal, Montreal, QC Canada; 11 Department of Pediatrics and Psychology, Université de Montréal, Montreal, QC Canada; 12 School of Public Health, University College Dublin, Belfield, Dublin, Ireland; 13 Department of Psychiatry and Addictology, Université de Montréal, Montreal, QC Canada; 14 Center for Studies in Behavioral Neurobiology, Concordia University, Montreal, QC Canada

**Keywords:** Risk factors, Biomarkers, Neuroscience

## Abstract

The excitatory neurotransmitter glutamate has been implicated in experience-dependent neuroplasticity and drug-seeking behaviors. Type 5 metabotropic glutamate (mGlu5) receptors might be particularly important. They are critically involved in synaptic plasticity and their availability has been reported to be lower in people with alcohol, tobacco, and cocaine use disorders. Since these reductions could reflect effects of drug use or pre-existing traits, we used positron emission tomography to measure mGlu5 receptor availability in young adults at elevated risk for addictions. Fifty-nine participants (age 18.5 ± 0.6) were recruited from a longitudinal study that has followed them since birth. Based on externalizing traits that predict future substance use problems, half were at low risk, half were at high risk. Cannabis use histories varied markedly and participants were divided into three subgroups: zero, low, and high use. Compared to low risk volunteers, those at elevated risk had lower [^11^C]ABP688 binding potential (BP_ND_) values in the striatum, amygdala, insula, and orbitofrontal cortex (OFC). Cannabis use by risk group interactions were observed in the striatum and OFC. In these regions, low [^11^C]ABP688 BP_ND_ values were only seen in the high risk group that used high quantities of cannabis. When these high risk, high cannabis use individuals were compared to all other participants, [^11^C]ABP688 BP_ND_ values were lower in the striatum, OFC, and insula. Together, these results provide evidence that mGlu5 receptor availability is low in youth at elevated risk for addictions, particularly those who frequently use cannabis.

## Introduction

In laboratory animals, type 5 metabotropic glutamate (mGlu5) receptors affect synaptic plasticity [1–3] and reward-related learning, including the acquisition and extinction of drug-seeking behaviors [4–9]. Based on these observations, individual differences in mGlu5 receptors have been proposed to influence susceptibility to substance use disorders (SUDs) [10–14]. Potentially worsening these effects, mGlu5 receptor expression, availability and signaling pathways can be altered following repeated exposure to addictive drugs, including amphetamine [10, 11], cocaine [12–15], alcohol [16] and cannabinoid receptor agonists [17]. The direction and magnitude of these effects appear to vary with the type of drug, extent of exposure, duration of abstinence and brain region. When low mGlu5 receptor levels develop in cortico-striatal-limbic regions, they might diminish the rewarding properties of some drugs [7, 18] and decrease the ability to adapt to changing environments [7, 18, 19]. This combination of effects might aggravate perseverative behaviors commonly seen in addictions [19–21].

In humans, the literature is smaller but positron emission tomography (PET) studies have provided evidence of altered striatal and corticolimbic mGlu5 availability in people with cocaine [22, 23], tobacco [24], and alcohol use disorders [25, 26], as compared to healthy volunteers. Since these differences could reflect pre-existing vulnerability traits, the effects of substance use, or some combination of the two, we used PET with [^11^C]ABP688 to measure mGlu5 receptor availability in emerging adults at varying risk for SUDs recruited from research participants who have been carefully characterized and followed since birth.

## Methods

### Participants

Fifty-nine volunteers (age 18.5 ± 0.6, range 18–20), living in the area of Montreal and Quebec City, Canada, were recruited from (i) the “Quebec Longitudinal Study of Child Development” (QLSCD; 572 members were born in 1996, *n* = 32 in current study [27]; 2120 were born in 1997–1998, *n* = 22 in current study [28] and (ii) the “Quebec Newborn Twin Study” (QNTS; 662 twin pairs born between 1995 and 1997, *n* = 5 in the current study [29]). Among the twins, only one sibling per pair was tested to avoid the confound of correlated traits. All participants in the present study had been followed since birth.

The neuroimaging participants were selected based on diverse adolescent externalizing (EXT) traits and behaviors (*e.g*., impulsivity, risk-taking and aggression) that predict future substance use problems [30–32]. These traits were measured annually between ages 10 and 16 years through self-report (QLSCD, *n* = 54) or teacher ratings (QNTS, *n* = 5) using the Social Behavior Questionnaire (SBQ) [33, 34]. Mean scores were calculated for the following SBQ subscales: hyperactivity, impulsivity, oppositional behavior, non-aggressive behavioral problems, physical aggression, proactive aggression, indirect aggression and reactive aggression. Composite EXT trait scores were aggregated using a minimum of two years’ data between 10 and 16 years. Cut-off values constituted the top and bottom 30% of EXT trait scores in the QLSCD participants born in 1996. Using these scores, half of the PET study participants were considered, a priori, at low risk for SUDs (Low EXT, *n* = 31). The other half were considered at elevated risk (High EXT, *n* = 28) (Table 1).

**Table 1:** Participant characteristics.

Characteristic	Low Risk (*n* = 31)			High Risk (*n* = 28)			Statistics	
	*N*	*M*	SD	*N*	*M*	SD	Test	*p* value
Age		18.4	0.6		18.6	0.6	*t*-test	0.23
Sex	20 f 11 m			16 f 12 m			Chi-square	0.56
Ethnicity	31 Caucasian			26 Caucasian, 1 Haitian, 1 Caucasian-Hispanic				
Years of education		12.1	0.6		11.7	1.3	*t*-test	0.13
Externalizing traits		0.5	0.3		2.4*	0.6	*t*-test	<0.001
AUDIT		4	2.6		5.9*	4.2	*t*-test	0.036
Alcohol age on onset	31	15.2	2.0	28	14.1	2.7	*t*-test	0.059
Alcohol, lifetime occasions	31	77	84	28	121	149	*t*-test	0.18
Alcohol, lifetime binges^a^	27	16	26	26	56*	89	*t*-test	0.03
							Chi-square	0.465
Cigarette smokers	2^b^			3^c^			Chi-square	0.56
Cannabis, lifetime occasions used	19	54	180	22	380*	580	*t*-test	0.016
							Chi-square	0.15
Cannabis, age of onset	19	16.2	1.2	22	15.5	1.5	*t*-test	0.093
Cannabis use within the past month	0			8*			Chi-square	0.001
Positive THC screen	0			5*			Chi-square	0.014
Amphetamine, lifetime occasions used	0			6*	65.8	112	*t*-test	0.19
							Chi-square	0.007
Cocaine, lifetime occasions used	2	4	4.2	8*	17.4	36.5	*t*-test	0.23
							Chi-square	0.024
MDMA, lifetime occasions used	2	6.5	4.9	9*	18.1*	37.1	*t*-test	0.014
							Chi-square	0.011
Psilocybin, lifetime occasion used	3	3.3	2.5	8	1.8	0.9	*t*-test	0.53
							Chi-square	0.063
LSD, lifetime occasions used	1	1		1	2		*t*-test	0.61
							Chi-square	0.94
Ketamine, lifetime occasions used	1	1		3	4	1	*t*-test	0.12
							Chi-square	0.25
GHB, lifetime occasions used	0			3	1.7	1.2	*t*-test	0.13
							Chi-square	0.06
Opiates, lifetime occasions used	0			1	2		*t*-test	0.33
							Chi-square	0.29
Drug Use (excluding cannabis), lifetime occasions	4	8.3	10.9	11*	66.6	131.4	*t*-test	0.14
							Chi-square	0.02
Current SUD	0			6^d^*			Chi-square	0.014
Current DSM-5 disorder other than SUD	0			5^e^*			Chi-square	0.007
Current or past DSM-5 disorder	1^f^			14^g^*			Chi-square	<0.001
SURPS Impulsivity	31	9.0	2.6	26	11.5*	2.9	*t-*test	0.001
SURPS Hopelessness	31	11.4	2.7	26	12.7	3.9	*t-*test	0.14
SURPS Anxiety sensitivity	31	9.5	3.1	26	10.2	2.3	*t-*test	0.31
SURPS Sensation Seeking	31	16.3	4.2	26	16.8	3.6	*t-*test	0.7
BIS Attention	31	14.1	3.4	26	16.4*	3.0	*t-*test	0.01
BIS Motor	31	18.3	3.1	26	21.6*	4.0	*t-*test	0.001
BIS Non-planning	31	22.0	4.2	26	25.2*	3.9	*t-*test	0.005
BIS total	31	54.5	7.8	26	63.2*	8.0	*t-*test	<0.001
SPSRQ Reward sensitivity	30	8.5	3.0	27	10.8*	4.5	*t-*test	0.03
SPSRQ Punishment sensitivity	30	9.6	4.8	27	11.9	5.0	*t-*test	0.08

*AUDIT* Alcohol Use Disorder Identification Test, *THC* tetrahydrocannabinol, *MDMA* 3,4-methyl enedioxy methamphetamine, *LSD* lysergic acid diethylamide, *GHB* gamma-hydroxybutyrate, *SUD* Substance Use Disorder, *FTND* Fagerström Test for Nicotine Dependence, *ADHD* Attention Deficit Hyperactivity Disorder, *SURPS* Substance Use Risk Profile Scale, *BIS* Barratt Impulsiveness Scale, *SPSRQ* Sensitivity to Punishment Sensitivity to Reward Questionnaire.

*significantly different from the low risk group using independent *t*-test with equal or unequal variance as appropriate or Chi-square, p < 0.05.

*t*-tests were run to test for differences in lifetime occasions including all participants. Chi-square was used to test for differences in proportion of individuals who ever used the substance.

^a^Four or more drinks for women, five or more for men.

^b^Two regular smokers (FTND > 2).

^c^One regular, two occasional social (FTND = 0) and two past smokers.

^d^Mild alcohol use disorder, *n* = 2; cannabis use disorder, *n* = 3; amphetamine use disorder, *n* = 1.

^e^ADHD, *n* = 1; ADHD and panic disorder, *n* = 1; dyslexia, *n* = 2; persistent depressive disorder, *n* = 1.

^f^Past major depressive disorder (MDD).

^g^Current and past ADHD, *n* = 1; dyslexia and past ADHD, *n* = 2; current persistent depressive disorder, *n* = 1; current mild alcohol use disorder (AUD), *n* = 1; current mild AUD and past mild binge eating disorder, *n* = 1; current mild cannabis use disorder, *n* = 1; current moderate cannabis use and past conduct disorder, *n* = 1; current cannabis use disorder, current panic disorder, current and past ADHD, past MDD and past AUD, *n* = 1; current moderate amphetamine use disorder and past moderate cannabis use disorder, *n* = 1; past MDD, *n* = 1; past ADHD, *n* = 1; past adjustment disorder with depressed mood, *n* = 1; past MDD, past adjustment disorder and past panic disorder, *n* = 1.

All participants were physically healthy and free of psychotropic medication. Five had been treated in the past for ADHD (methylphenidate alone or in combination with atomoxetine). None had used these medications in the previous 2 years, and both duration of use (mean: 5.6 ± 3.3 years, range 1–10 years) and time since last use (4.2 ± 2.6 years, range 2–7 years) varied widely. Four participants were in the high risk group and one was in the low risk group. Among these, one was in the zero cannabis use group, two were in the low cannabis use group and two were in the high cannabis group. Two participants had received treatment for migraines (*n* = 1, tricyclic antidepressant, amitriptyline, Elavil; *n* = 1, hydromorphone, Dilaudid). One was in the high risk, high cannabis use group; the other in the high risk, low cannabis use group. For both, medication use was sporadic, taken on an as-need basis for a brief period only (1 week and 1 month respectively). Neither participant took the medication during the month prior to testing. Five were current tobacco smokers, as determined by self-reported use of at least one cigarette per week. Among these five smokers, two were occassional social smokers (Fagerström Test for Nicotine Dependence or FTND Score = 0, [35]) and three smoked more frequently (FTND range 2–5, 6–10 cigarettes per day). Out of 59 participants, six met criteria for a current SUD (mild alcohol use disorder, *n* = 2; cannabis use disorder, *n* = 3; amphetamine use disorder, *n* = 1) and five met criteria for a current non-substance related disorder (dyslexia, *n* = 2; persistent depressive disorder, *n* = 1; ADHD, *n* = 1; ADHD and panic disorder, *n* = 1) as determined by the Structured Clinical Interview for DSM-5 [36]. Five other participants met criteria for a past DSM-5 disorder only (Table 1, S1).

Before the PET session, participants abstained from caffeine for at least four hours, from alcohol for at least 24 h and from nicotine and cannabis for at least 12 h, with the exception of one participant who smoked half a cigarette and took one puff of cannabis 4.5 h prior to the PET scan. Urine drug screens and pregnancy tests were obtained prior to the PET session (Express Diagnostics, MN, USA), and participants were excluded if they tested positive for pregnancy or any ‘recreational’ drug other than cannabis (amphetamine, benzodiazepines, buprenorphine, cocaine, 3,4-methylenedioxy-methamphetamine (MDMA), methamphetamine, methadone, or opioids). The urine drug screen sensitivity varies depending on the drug. For daily and near daily cannabis users, positive test results can appear for up to 25 days of abstinence [37]. Females who were not using hormonal contraceptives (*n* = 17 out of 36) were tested during the follicular phase of their menstrual cycle (self-report). All participants provided written informed consent. The study was carried out in accordance with the Declaration of Helsinki, and approved by the Research Ethics Board of the Montreal Neurological Institute, McGill University, and the ethics committee of the CHU Sainte-Justine Research Center.

### Substance use & personality measures

**Fig. 1:**
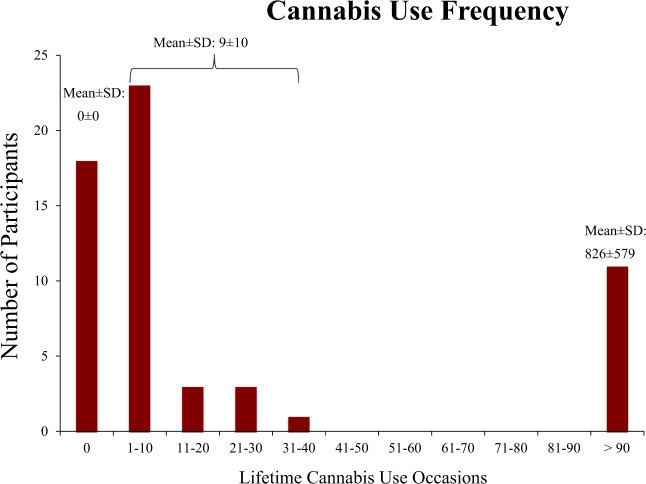
The trimodal distribution of lifetime cannabis use occasions. Distribution of number of participants and cannabis use frequency (mean ± SD) for the three cannabis use groups: 1) zero lifetime cannabis use; 2) low cannabis use, between 1 and 40 lifetime uses; and 3) high cannabis use or > 90 lifetime uses.

Drug and alcohol use data were collected prospectively during annual interviews throughout adolescence (age 11–16). These data were augmented by information collected during the index interview (see above). This included administration of the Alcohol Use Disorder Identification Test (AUDIT) [38] and a time-line follow back interview [39] for lifetime use of alcohol, cannabis, amphetamine, MDMA, cocaine, psilocybin, lysergic acid diethylamide, ketamine, and opiates. For alcohol use frequency, we collected information on lifetime number of alcohol use occasions and occasions of binge drinking. Binge drinking refers to occasions where participants ingested more than four (women) or five (men) drinks. Since substance use for drugs other than cannabis was low and only present in a small subset of individuals (Table 1), we calculated the sum of lifetime frequencies for all illicit substances other than cannabis for each participant. Cannabis use was highly variable and exhibited a trimodal distribution (Fig. 1). We therefore divided participants into three groups: (1) zero lifetime cannabis use (*n* = 18); (2) low cannabis use, indicating between one and 40 lifetime uses (*n* = 30); and (3) high cannabis use, reflecting more than 90 lifetime uses (*n* = 11). Tobacco smoking status included two levels, defined as those who are currently smoking (group 1) or not (group 2). Group 1 included both occassional (FTND = 0) and more frequent (FTND ≥ 2) smokers.

Participants also completed the Substance Use Risk Profile Scale (SURPS) [40], Barratt Impulsiveness Scale (BIS-11) [41], and Sensitivity to Punishment (SP) and Sensitivity to Reward (SR) Questionnaire (SPSRQ) [42]. The scales have demonstrated validity in adolescents and young adults and have acceptable test-retest reliability [42–46].

### PET image acquisition

PET scans were acquired using a Siemens ECAT high-resolution research tomograph (with a spatial resolution range between 2.3–3.4 mm full width at half maximum). All scans started between 11:00am and 1:00 pm, with the exception of one scan that started at 3:00 pm. There were no risk group (*p* > 0.4) or subgroup (*p* > 0.5) differences in scan start times. A 6-minute ^137^Cs transmission scan was acquired for attenuation correction, followed by an average bolus injection of 367 ± 30MBq [^11^C]ABP688, which corresponds to an average radiation exposure of 1.4 mSv. The average injected mass and specific activity were 10.1 ± 6.3 µg and 26.4 ± 34.3GBq/μmol. A significant difference was observed in injected mass (low risk: 7.0 ± 6.2 µg; high risk: 12.4 ± 5.9 µg) and specific activity (low risk: 37.9 ± 40.4; high risk:  13.7 ± 19.7 GBq/μmol) between high and low risk individuals. Additional analyses were conducted with injected mass and specific activity as covariates. Following the [^11^C]ABP688 scan, the same participants underwent a 90-min [^18^F]fallypride scan. These results are presented elsewhere [47]. Dynamic data were collected and reconstructed as previously described [23]. In brief, data were acquired over 60-min in list mode in 26 timeframes of progressively increasing duration (frame duration: 6 × 30 s, 4 × 60 s, 8 × 120 s, 3 × 240 s, 5 × 300 s) and reconstructed using an ordered subset maximization algorithm. Reconstruction included correction for motion, random events, attenuation, scatter, decay and intensity normalization. No task was administered and participants were instructed to remain awake and rest quietly.

### Magnetic resonance imaging

High-resolution (1 mm isotropic voxel) T1-weighted magnetic resonance imaging (MRI) scans were acquired on each participant for anatomical co-registration using a 3T Siemens Trio TIM scanner (MPRAGE sequence, repetition time = 2300 ms, echo time = 3.42 ms, flip angle = 9°, field of view = 256 mm and matrix = 256 × 256).

### Behavioral analyses

High versus low risk groups were compared on behavioral and demographic measures using independent t-tests or Chi-Square tests (Table 1). Multivariate ANOVAs were conducted to assess group differences on the SURPS, BIS-11 and SPSRQ.

### Image analysis

[^11^C]ABP688 non-displaceable binding potential (BP_ND_) values were computed relative to nonspecific binding in cerebellar gray matter using the simplified reference tissue model (SRTM) [48], which shows high correspondence (R^2^ = 0.94) with values derived from 2-tissue compartment modeling methods using arterial sampling [49].

### ROI analysis

Each MRI was pre-processed with the CIVET pipeline version 2.0.0 (wiki.bic.mni.mcgill.ca/ServicesSoftware/CIVET), which included correction for image intensity and non-uniformity, and a non-linear and linear transformation to standardized stereotaxic space using the ICBM template [50]. The normalized images were then classified into white matter, gray matter and cerebral spinal fluid, and automatically segmented using a probabilistic atlas based approach (Automatic Nonlinear Image Matching and Anatomical Labeling or ANIMAL) [51]. Regions of interest (ROIs) including the prefrontal cortex (medial orbitofrontal cortex, mOFC; lateral orbitofrontal cortex, lOFC; medial frontal cortex, mPFC), insula and subcortical limbic regions (amygdala, hippocampus) were defined using this segmentation. The ROIs in the striatum were also defined on each individual’s MRI in stereotaxic space. They were based on the functional segmentation proposed by Mawlawi et al. [52], and included the ventral limbic striatum (VS), associative striatum (AST) and somatosensory striatum (SMST). ROI masks were applied to each summed radioactivity PET image using nonlinear registration. Time-activity curves were extracted from each ROI in native PET space using tools developed by the Turku PET Centre (http://www.turkupetcentre.net/). For each ROI, BP_ND_ values were calculated using SRTM.

### Voxel-wise analysis

Voxel-wise BP_ND_ maps were created in native space, then co-registered in MNI space and smoothed using a 5.7 mm full width half maximum Gaussian filter.

### Statistical analysis

#### ROI analysis

Analysis of covariance (ANCOVA) was used to test for the effects of risk and cannabis use on BP_ND_ values in different ROIs using SPSS version 24. A repeated measures ANCOVA was conducted with all ROIs (VS, AST, SMST, mOFC, lOFC, mPFC, amygdala, hippocampus and insula) as within subject factors, and EXT risk group and cannabis use group as between-subject factors. Follow-up univariate ANCOVAs and least significant difference *post hoc* tests were performed for each individual ROI when a main effect of group or a group by region interaction was found. For all ANCOVAs and *t*-tests, sex, isomer ratio, AUDIT scores, tobacco smoking status and drug use (other than cannabis) were included as covariates. Each of these factors is associated with differences in [^11^C]ABP688 BP_ND_ [22–24, 26, 53, 54]. Greenhouse-Geisser corrections were applied when the assumption of sphericity was violated (*p* < 0.05).

Independent *t*-tests were performed to compare BP_ND_ values in heavy cannabis users in the high EXT group (*n* = 9) *vs*. all low EXT individuals (*n* = 31). All tests used marginal means controlled for individual differences in sex, isomer ratio, AUDIT scores, smoking status and drug use (other than cannabis).

#### Voxel-wise whole brain analysis

Whole brain voxel-wise analyses were conducted using SPM12. *T*-maps were generated to compare BP_ND_ values in high risk/high cannabis users *vs*. all other individuals, controlling for individual differences in sex, isomer ratio, AUDIT scores, tobacco smoking status, and drug use (other than cannabis). Significant differences are reported at *p* < 0.05, family-wise error (FWE) corrected for the whole brain (peak or cluster level; cluster-level analyses were based on a primary voxel-level threshold of *p* < 0.001).

## Results

### Participant characteristics

The high vs. low risk groups were well matched for age, sex and years of education, and by design, differed on EXT trait scores (*t* = 16.02, *p* < 0.001). They also differed on EXT related features as measured by the SURPS (Wilk’s lambda: F(4, 52) = 3.11, *p* = 0.023), BIS-11 (Wilk’s lambda: F(3,53) = 5.72, *p* = 0.002) and SPSRQ (Wilk’s lambda: F(2,54) = 3.63, *p* = 0.033). Compared to low risk participants, the high risk group scored higher on levels of impulsivity (BIS-11: F(1, 55) = 17.43, *p* < 0.000; SURPS: F(1,55)=12.1, *p* = 0.001) and reward sensitivity (SPSRQ reward: F(1, 55) = 5.22, *p* = 0.029).

As expected, the high risk individuals reported more lifetime occasions of binge drinking (*t* = 2.28, *p* = 0.03) and alcohol-related problems (*t* = 2.15, *p* = 0.036), had used more cannabis (*t* = 2.54, *p* = 0.016) and MDMA (*t* = 2.56, *p* = 0.014), and were more likely to have tried various stimulant drugs (amphetamine: Chi = 7.4, (df = 1,59), *p* = 0.007; cocaine: Chi = 5.1, (df = 1,59), *p* = 0.024; MDMA: Chi = 6.4, (df = 1,59), *p* = 0.01) (see Table 1). On the day of the PET scan, more individuals in the high risk than low risk group had used cannabis within the past month (Chi = 10.3, (df = 1,59), *p* = 0.001), and tested positive for delta-9-tetrahydrocannabinol (THC) (Chi = 6.1, (df = 1,59), *p* = 0.014) on the urine toxicology screen. All five participants with a positive THC screen were in the high risk, high cannabis using group. Out of eight participants who had used cannabis within the past month, seven were in the high risk, high cannabis using group. Significantly more individuals in the high risk group met criteria for a current SUD (Chi = 7.4, (df = 1,59), *p* = 0.007) and for neuropsychiatric disorders other than SUDs (Chi = 6.05, (df = 1,59), *p* = 0.014) when compared to the low risk group.

### Region of interest analyses

The omnibus rmANCOVA for [^11^C]ABP688 BP_ND_ values yielded a significant main effect of risk group (F(1,48) = 4.65, *p* = 0.036, η^2^ = 0.088) and two-way risk group x cannabis use (F(2,48) = 3.38, *p* = 0.042, η^2^ = 0.124) and risk group x ROI interactions (F(4.14, 199) = 4.40, *p* = 0.002, partial η^2^ = 0.084) but not a three-way risk group x cannabis use x ROI interaction (F(8.27,199) = 1.73, *p* = 0.091, η^2^ = 0.067).

Follow-up univariate ANCOVAs for each ROI yielded main effects of risk group reflecting significantly lower BP_ND_ values in the high compared to the low risk group in all subregions of the striatum (VS: (F(1,48) = 7.0, *p* = 0.011, η^2^ = 0.127; AST: F(1,48) = 6.69, *p* = 0.013, η^2^ = 0.122; SMST: F(1,48) = 5.50, *p* = 0.023, η^2^ = 0.103), the lateral (F(1,48) = 4.48, *p* = 0.039, η^2^ = 0.085) and medial OFC (F(1,48) = 4.25, *p* = 0.045, η^2^ = 0.081), the amygdala (F(1,48) = 5.24, *p* = 0.027, η^2^ = 0.098) and the insula (F(1,48) = 4.68, *p* = 0.035, η^2^ = 0.089).

**Fig. 2:**
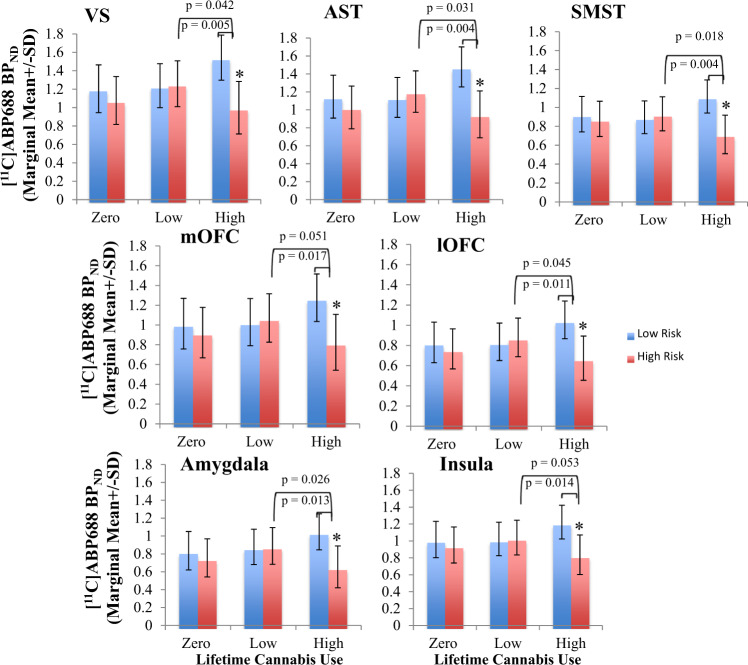
Lower [^11^C]ABP688 BP_ND_ in high EXT risk, high cannabis users: ROI analysis. [^11^C]ABP688 BP_ND_ values in cortical and subcortical limbic regions in high and low EXT risk individuals with zero (low EXT risk *n* = 12; high EXT risk *n* = 6), low (low EXT risk *n* = 17; high EXT risk *n* = 13) and high (low EXT risk *n* = 2; high EXT risk *n* = 9) frequencies of lifetime cannabis use occasions. Mean values are adjusted for sex, isomer ratio, AUDIT scores, tobacco smoking status and drug use other than cannabis. VS ventral striatum, AST associative striatum, SMST somatosensory striatum, mOFC medial orbitofrontal cortex, lOFC lateral orbitofrontal cortex. *significantly different from all low EXT risk groups, *p* < 0.05.

Significant risk group x cannabis use interactions were observed in all subregions of the striatum (VS: F(2,48) = 3.82, *p* = 0.029, η^2^ = 0.137; AST: F(2,48) = 5.15, *p* = 0.009, η^2^ = 0.177; SMST: F(2,48) = 4.37, *p* = 0.018, η^2^ = 0.154) and the lOFC (F(2, 48) =3.64), *p* = 0.034, η^2^ = 0.132) and approached significance in the mOFC (F(1,48) = 3.01, *p* = 0.059, η^2^ = 0.112). In all regions, the interactions reflected significantly lower BP_ND_ values in the high risk, high cannabis users (Fig. 2 and Table S2). The same pattern of effects was seen in the other ROIs (Fig. 2 and Table S2). When the high risk, high cannabis use individuals (*n* = 9) were compared to all low risk participants (*n* = 31), [^11^C]ABP688 binding values were significantly lower in the striatum (all subregions *p* < 0.02), OFC (all subregions, *p* < 0.05), amygdala (*p* < 0.02) and insula (*p* < 0.05).

### Brain wide voxelwise analyses

**Fig. 3:**
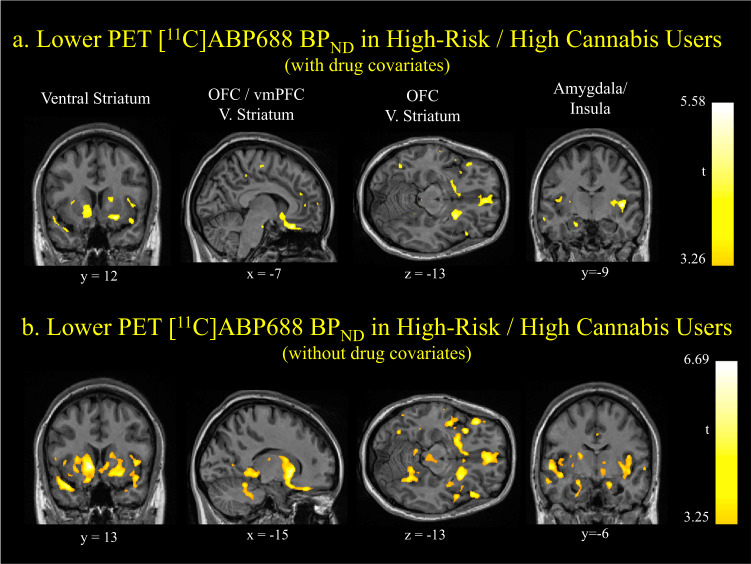
Lower [^11^C]ABP688 BP_ND_ in high EXT risk, high cannabis users: whole brain voxel-wise analysis. **a** *T*-maps illustrating significantly lower [^11^C]ABP688 BP_ND_ in high EXT risk, high cannabis users (*n* = 9) compared to all other participants (*n* = 50) in mOFC, ventral striatum and insula with other substance use (AUDIT, smoking status and drug use other than cannabis), sex and *E*-isomer ratio as covariates. Cluster-level *ps* < 0.05, FWE corrected. **b** *T*-maps illustrating significantly lower [^11^C]ABP688 BP_ND_ in high EXT risk, high cannabis users (*n* = 9) compared to all other participants (*n* = 50) in mOFC, ventral striatum and insula without other substance use as covariates (AUDIT, smoking status and drug use other than cannabis). Sex and *E*-isomer ratio were included as covariates. Peak-level *ps* < 0.05, FWE corrected.

Voxelwise analyses (also controlling for the use of tobacco, alcohol, all other drugs, sex, and [^11^C]ABP688 *E-*isomer percent) were consistent with the ROI findings. BP_ND_ values were significantly lower in high risk, high cannabis use individuals (*n* = 9) compared to all other 50 participants in the mOFC, ventral striatum and insula (cluster-level ps<0.05, FWE corrected, cluster size > 1500 voxels, Fig. 3a). These group differences were larger without the inclusion of other substance use covariates (Fig. 3b), with particularly robust effects in the bilateral ventral striatum (peak-level *p* = 0.003, FWE corrected), medial OFC (peak-level *p* = 0.024, FWE corrected), left lateral OFC (peak-level *p* = 0.018, FWE corrected) and insula (peak-level *p* = 0.003, FWE corrected).

The pattern of results remained unchanged when the measures of alcohol use frequency were included as covariates instead of AUDIT scores and when excluding the participant who smoked cannabis 4.5 h prior to the scan, the person whose scan started late (3pm vs. 11am-1pm) or the two participants who had previously taken medication sporadically for the relief of migraines. Adding injected mass and specific activity of the tracer or past ADHD medication as an additional covariate to the model did not change the results. Injected mass did not differ between the three high risk subgroups (*p* > 0.4), despite the fact that low mGlu5 receptor availability was seen in the high-risk, high cannabis using subgroup only.

## Discussion

The present study identified two novel contributors to low mGlu5 receptor availability: high EXT risk traits for SUDs and heavy cannabis use. Since high EXT youth are more likely to use large quantitites of cannabis, these features are intertwined. And yet there was some evidence that the factors make separable contributions. BP_ND_ values were not altered in high EXT participants who did not use large quantities of cannabis, nor was there a main effect of cannabis use. Instead, BP_ND_ values were lowest in participants with both high EXT traits and high cannabis use.

The low mGlu5 receptor availabilities were seen within cortical-subcortical limbic regions. These circuits have also dominated results in studies of people with current SUDs [22–26]. Although smaller differences might be observed elsewhere, it is noteworthy that the most prominent differences in mGlu5 receptor availability occur within circuits that regulate approach-avoidance behaviors and learning about emotionally relevant events [21, 55]. By diminishing reward-related learning, low mGlu5 transmission might narrow behavioral repertoires, aggravating the development of problematic substance use [7, 18–21].

The decreases in mGlu5 receptor availability might be caused, at least in part, by the pharmacological effects of cannabis use. In laboratory animals, extensive CB1 activation during adolescence can decrease mGlu5 protein levels in the adult [17]. These effects are reciprocal. Lowered mGlu5 receptor levels decrease synthesis of the endogenous CB1 agonist, 2-arachnidonoylglycerol [17]. Together, these results suggest that extensive adolescent cannabis exposure could cause long-term reductions in mGlu5 transmission, leading to reduced synaptic plasticity, both directly and indirectly, through functional interactions between mGlu5 and endocannabinoid signaling [1, 2].

The above possibility noted, the present study did not identify a main effect of cannabis use alone. Instead, there were main effects of risk (striatum, amygdala, insula, OFC) and risk x cannabis use interactions (striatum, OFC). Even in the regions without a statistically significant risk x cannabis use interaction, visual inspection of the data indicated that values were especially low in the high risk, high cannabis users. These observations raise the possibility that low mGlu5 availability could be a pre-existing trait that is aggravated by cannabis use or a trait that is best identified by the combination of cannabis use plus EXT features. Of potential relevance for both interpretations, variants of the gene that encodes for mGlu5 affect risk for SUDs [56], and we have recently observed preliminary evidence that elevated mGlu5 levels might protect people from drug-induced behavioral sensitization [11].

Previous studies have identified evidence of altered mGlu5 receptor availablity in people with current cocaine [22, 23], alcohol [25, 26] and tobacco use disorders when compared with healthy volunteers [24]. The reductions in [^11^C]ABP688 binding seen in tobacco smokers [24] return to normal levels following a period of extended abstinence [57], suggesting that, in this population, low mGlu5 levels are not a pre-existing trait. In comparison, recently abstinent heavy cocaine users exhibit low [^11^C]ABP688 binding values [22, 23] and these decrements appear to become larger over a two-week period without cocaine [23]. In this latter study [23], we also found that we could disentangle the influence of tobacco use but not cannabis. The effects in people with an alcohol use disorder have been more variable, with evidence of both increased [25] and decreased [26] mGlu5 receptor availability reported. The authors of the former study specifically recruited participants who did not use either tobacco or cannabis [25]. If cannabis use was more prominent in the latter study [26], one speculative interpretation is that tobacco and cannabis decrease mGlu5 receptor levels while alcohol use alone increases mGlu5 receptor availability [16]. This will require further study.

### Limitations

The observations reported here should be considered in light of the following. The primary *a priori* hypothesis was that mGlu5 receptor levels would be lower in people at risk for substance use problems. This was seen, particularly within the striatum, amygdala, OFC, and insula. The interaction between risk and cannabis use was followed up based on the striking trimodal distribution of cannabis use. However, the result is compelling. The interaction effect was seen in four ROIs, CB1 stimulation decreases mGlu5 levels in laboratory animals [17], and, in the present study, lower [^11^C]ABP688 binding was consistently observed in high risk individuals who were using largest amounts of cannabis. Second, the overall sample size is large for a PET study (*n* = 59), but the subgroups are smaller. This noted, the main effect of group was seen when comparing sample sizes of 28 *vs*. 31 while the low BP_ND_ values in high risk heavy cannabis users was evident when comparing 9 *vs*. 31 and 9 *vs*. 50. Third, the high *vs*. low risk participants exhibited different patterns of cannabis use. The potential influence of these features will require further study. Fourth, injected tracer mass differed between high and low risk individuals, however adding this as a covariate to our model did not change the result and injected mass did not differ between the three high risk subgroups (*p* > 0.4) despite the fact that low [^11^C]ABP688 binding values were seen in the high-risk, high cannabis using subgroup only. Fifth, [^11^C]ABP688 binding potential in humans can exhibit high levels of variability, and the use of a cerebellar reference region instead of arterial blood sampling may have contributed to this. However, this variability would have decreased rather than increased the probability of observing group differences. More importantly, the use of the cerebellum as a reference region has been validated based on a high correspondance between BP_ND_ values and k3/k4 kinetic constants ratio estimates (R^2^ > 0.93) derived with the arterial input function [49]. Indeed, despite the presence of mGlu5 receptors in the cerebellum, only a negligible fraction specifically binds to the radiotracer [58], minimizing the likelihood of a bias or difference in cerebellar uptake between our groups. Instead, we recently documented that much of the variability in [^11^C]ABP688 BP_ND_ [59] reflects effects of time of day [60], *(Z)-*isomer content in the synthesized batch of tracer [53], and sex [54]. All three factors were controlled for in the present study. Sixth, group differences in [^11^C]ABP688 binding values were larger when other substance use was not included as a covariate. This suggests that mGlu5 receptor availability is especially low in people at elevated risk for addictions with the effect propelled by the influence of multiple substances. The exact contribution of these multiple substances is likely to be complex. When trying to address this statistically, covariates can increase statistical power by removing noise in the data and reduce statistical power by decreasing degrees of freedom. Because of these effects, it is recommended that covariates should be included only when there are *a priori* reasons to do so [61]. This approach was used here. Finally, the substance use and other behavioral history features were obtained by self-report. However, the participants have been followed since birth providing greater confidence than is possible from retrospective reports about the distant past.

## Funding and disclosure

This work was supported by grants from the Canadian Institutes for Health Research MOP-133537 (ML, JRS, CB and MB), MOP-44072 (JRS) and MOP-97910 (JRS, SP); from the Fonds de Recherche du Quebec - Sante numbers 981055, 991027 (JRS) and 35282 (NCR); Fonds de Recherche du Quebec - Societe et Culture numbers 2002-RS-79238, 2009-RG-124779 (JRS and MB); and from the Social Sciences and Humanities Research Council of Canada numbers 410-99-1048 and 839-2000-1008 (JRS and MB). ML and CB were recipients of McGill University research chairs, while salary awards were provided by CIHR to NJ and NCR and by FRQS to MT. The QLSCD cohort born 1997-1998 is led by the Institut de la Statistique du Québec in collaboration with several departments and agencies of the Government of Quebec and many collaborating researchers including the authors of this article. Project completion with QLSCD respondents was authorized by the QLSCD Steering Committee. The authors declare no conflict of interest.

## Supplementary information

Supplemental material

## References

[1] Robbe D, Kopf M, Remaury A, Bockaert J, Manzoni OJ (2002). Endogenous cannabinoids mediate long-term synaptic depression in the nucleus accumbens. Proc Natl Acad Sci USA.

[2] Olmo IG, Ferreira-Vieira TH, Ribeiro FM (2016). Dissecting the signaling pathways Involved in the crosstalk between metabotropic glutamate 5 and cannabinoid type 1 receptors. Mol Pharmacol..

[3] Buschler A, Manahan-Vaughan D (2017). Metabotropic glutamate receptor, mGlu5, mediates enhancements of hippocampal long-term potentiation after environmental enrichment in young and old mice. Neuropharmacology..

[4] Bird MK, Lohmann P, West B, Brown RM, Kirchhoff J, Raymond CR (2014). The mGlu5 receptor regulates extinction of cocaine-driven behaviours. Drug Alcohol Depend.

[5] Kenny PJ, Boutrel B, Gasparini F, Koob GF, Markou A (2005). Metabotropic glutamate 5 receptor blockade may attenuate cocaine self-administration by decreasing brain reward function in rats. Psychopharmacol (Berl).

[6] O’Connor EC, Crombag HS, Mead AN, Stephens DN (2010). The mGluR5 antagonist MTEP dissociates the acquisition of predictive and incentive motivational properties of reward-paired stimuli in mice. Neuropsychopharmacology..

[7] Szumlinski KK, Shin CB (2018). Kinase interest you in treating incubated cocaine-craving? A hypothetical model for treatment intervention during protracted withdrawal from cocaine. Genes Brain Behav.

[8] Kumaresan V, Yuan M, Yee J, Famous KR, Anderson SM, Schmidt HD (2009). Metabotropic glutamate receptor 5 (mGluR5) antagonists attenuate cocaine priming- and cue-induced reinstatement of cocaine seeking. Behav Brain Res.

[9] Chiamulera C, Epping-Jordan MP, Zocchi A, Marcon C, Cottiny C, Tacconi S (2001). Reinforcing and locomotor stimulant effects of cocaine are absent in mGluR5 null mutant mice. Nat Neurosci..

[10] Szumlinski KK, Lominac KD, Campbell RR, Cohen M, Fultz EK, Brown CN (2017). Methamphetamine addiction vulnerability: the glutamate, the bad, and the ugly. Biol Psychiatry..

[11] Smart K, Nagano-Saito A, Milella M, Sakae DY, M. F, Vigneault E, et al. Low metabotropic glutamate type 5 receptor binding is associated with d-amphetamine sensitization in mice and humans. J Psychiatry Neurosci. 2020. in press. 10.1503/jpn.190162 PMC7955855 32559027

[12] de Laat B, Weerasekera A, Leurquin-Sterk G, Bormans G, Himmelreich U, Casteels C (2018). Glutamatergic biomarkers for cocaine addiction: a longitudinal study using MR spectroscopy and mGluR5 PET in self-administering rats. J Nucl Med.

[13] Hao Y, Martin-Fardon R, Weiss F (2010). Behavioral and functional evidence of metabotropic glutamate receptor 2/3 and metabotropic glutamate receptor 5 dysregulation in cocaine-escalated rats: factor in the transition to dependence. Biol Psychiatry..

[14] Knackstedt LA, Moussawi K, Lalumiere R, Schwendt M, Klugmann M, Kalivas PW (2010). Extinction training after cocaine self-administration induces glutamatergic plasticity to inhibit cocaine seeking. J Neurosci..

[15] Ben-Shahar O, Obara I, Ary AW, Ma N, Mangiardi MA, Medina RL (2009). Extended daily access to cocaine results in distinct alterations in Homer 1b/c and NMDA receptor subunit expression within the medial prefrontal cortex. Synapse..

[16] Campbell RR, Domingo RD, Williams AR, Wroten MG, McGregor HA, Waltermire RS (2019). Increased alcohol-drinking induced by manipulations of mGlu5 phosphorylation within the bed nucleus of the stria terminalis. J Neurosci..

[17] Gleason KA, Birnbaum SG, Shukla A, Ghose S (2012). Susceptibility of the adolescent brain to cannabinoids: long-term hippocampal effects and relevance to schizophrenia. Transl Psychiatry..

[18] Olive MF (2010). Cognitive effects of Group I metabotropic glutamate receptor ligands in the context of drug addiction. Eur J Pharm.

[19] Bird MK, Lawrence AJ (2009). The promiscuous mGlu5 receptor-a range of partners for therapeutic possibilities?. Trends Pharm Sci.

[20] Smart K, Scala S, El Mestikawy S, Benkelfat C, Leyton M. Cocaine addiction and metabotropic receptor subtype 5. In: Preddy VR, editor The neuroscience of cocaine: mechanisms and treatment. London, UK: Academic Press; 2017. p. 269-78.

[21] Leyton M, Vezina P (2014). Dopamine ups and downs in vulnerability to addictions: a neurodevelopmental model. Trends Pharm Sci.

[22] Martinez D, Slifstein M, Nabulsi N, Grassetti A, Urban NB, Perez A (2014). Imaging glutamate homeostasis in cocaine addiction with the metabotropic glutamate receptor 5 positron emission tomography radiotracer [11C]ABP688 and magnetic resonance spectroscopy. Biol Psychiatry..

[23] Milella MS, Marengo L, Larcher K, Fotros A, Dagher A, Rosa-Neto P (2014). Limbic system mGluR5 availability in cocaine dependent subjects: a high-resolution PET [11C]ABP688 study. Neuroimage..

[24] Akkus F, Ametamey SM, Treyer V, Burger C, Johayem A, Umbricht D (2013). Marked global reduction in mGluR5 receptor binding in smokers and ex-smokers determined by [11C]ABP688 positron emission tomography. Proc Natl Acad Sci USA.

[25] Akkus F, Mihov Y, Treyer V, Ametamey SM, Johayem A, Senn S (2018). Metabotropic glutamate receptor 5 binding in male patients with alcohol use disorder. Transl Psychiatry..

[26] Leurquin-Sterk G, Ceccarini J, Crunelle CL, de Laat B, Verbeek J, Deman S (2018). Lower limbic metabotropic glutamate receptor 5 availability in alcohol dependence. J Nucl Med.

[27] Jetté M, Desrosiers H, Tremblay RE. Survey of 5 months old infants: preliminary report from the Québec Longitudinal Study of Childhood Development (QLSCD). Montréal, QC, Canada. Ministère de La Santé et Des Services Sociaux. Gouvernement Du Québec, 1998.

[28] Jetté M, Des Groseilliers L (2000). Survey description and methodology of the Longitudinal Study of Child Development in Québec (ELDEQ 1998-2002).

[29] Boivin M, Brendgen M, Dionne G, Dubois L, Perusse D, Robaey P (2013). The Quebec Newborn Twin Study into adolescence: 15 years later. Twin Res Hum Genet.

[30] Edwards AC, Gardner CO, Hickman M, Kendler KS (2016). A prospective longitudinal model predicting early adult alcohol problems: evidence for a robust externalizing pathway. Psychol Med.

[31] Pingault JB, Cote SM, Galera C, Genolini C, Falissard B, Vitaro F (2013). Childhood trajectories of inattention, hyperactivity and oppositional behaviors and prediction of substance abuse/dependence: a 15-year longitudinal population-based study. Mol Psychiatry.

[32] Foster KT, Hicks BM, Zucker RA (2018). Positive and negative effects of internalizing on alcohol use problems from childhood to young adulthood: the mediating and suppressing role of externalizing. J Abnorm Psychol.

[33] Behar L, Stringfield S (1974). A behavior rating scale for the preschool child. Developmental Psychol.

[34] Tremblay RE, Vitaro F, Gagnon C, Piché C, Royer N (1992). A prosocial scale for the preschool behaviour questionnaire: concurrent and predictive correlates. Int J Behav Dev.

[35] Heatherton TF, Kozlowski LT, Frecker RC, Fagerstrom KO (1991). The Fagerstrom Test for Nicotine Dependence: a revision of the Fagerstrom Tolerance Questionnaire. Br J Addiction.

[36] First MB, Williams JBW, Karg RS, Spitzer RL (2015). Structured Clinical Interview for DSM-5, Research Version (SCID-5 for DSM-5, Research Version; SCID-5-RV).

[37] Verstraete AG (2004). Detection times of drugs of abuse in blood, urine, and oral fluid. Ther Drug Monit.

[38] Saunders JB, Aasland OG, Babor TF, de la Fuente JR, Grant M (1993). Development of the Alcohol Use Disorders Identification Test (AUDIT): WHO collaborative project on early detection of persons with harmful alcohol consumption-II. Addiction..

[39] Sobell LC, Sobell MB. Timeline follow-back: A technique for assessing self-reported alcohol consumption. In: Litten RZ, Allen JP, editors. Measuring alcohol consumption: psychosocial and biochemical methods. Totowa, NJ, US: Humana Press; 1992. p. 41–72.

[40] Woicik PA, Stewart SH, Pihl RO, Conrod PJ (2009). The Substance Use Risk Profile Scale: a scale measuring traits linked to reinforcement-specific substance use profiles. Addictive Behav..

[41] Patton JH, Stanford MS, Barratt ES (1995). Factor structure of the Barratt impulsiveness scale. J Clin Psychol.

[42] Torrubia R, Avila C, Molto J, Caseras X (2001). The Sensitivity to Punishment and Sensitivity to Reward Questionnaire (SPSRQ) as a measure of Gray’s anxiety and impulsivity dimensions. Pers Indiv Differ.

[43] Vandeweghe L, Matton A, Beyers W, Vervaet M, Braet C, Goossens L (2016). Psychometric properties of the BIS/BAS scales and the SPSRQ in Flemish adolescents. Psychol Belg.

[44] Castellanos-Ryan N, O’Leary-Barrett M, Sully L, Conrod P (2013). Sensitivity and specificity of a brief personality screening instrument in predicting future substance use, emotional, and behavioral problems: 18-month predictive validity of the Substance Use Risk Profile Scale. Alcohol, Clin Exp Res.

[45] Castonguay-Jolin L, Perrier-Menard E, Castellanos-Ryan N, Parent S, Vitaro F, Tremblay RE (2013). SURPS French version validation in a Quebec adolescent population. Can J Psychiatry.

[46] Stanford MS, Mathias CW, Dougherty DM, Lake SL, Anderson NE, Patton JH (2009). Fifty years of the Barratt Impulsiveness Scale: an update and review. Pers Indiv Differ.

[47] Jaworska N, Cox SML, Tippler M, Castellanos-Ryan N, Benkelfat C, Parent S, et al. Extra-striatal D2/3 receptor availability in youth at risk for addiction. Neuropsychopharmacology. 2020. 10.1038/s41386-020-0662-7. 10.1038/s41386-020-0662-7 PMC7360619 32259831

[48] Lammertsma AA, Hume SP (1996). Simplified reference tissue model for PET receptor studies. Neuroimage.

[49] Milella MS, Reader AJ, Albrechtsons D, Minuzzi L, Soucy JP, Benkelfat C, et al. Human PET validation study of reference tissue models for the mGluR5 ligand [11C]ABP688. 41st Annual Meeting of the Society for Neuroscience, Washington, DC, 2011.

[50] Sled JG, Zijdenbos AP, Evans AC (1998). A nonparametric method for automatic correction of intensity nonuniformity in MRI data. IEEE Trans Med Imaging.

[51] Collins DL, Evans AC (1997). Animal: validation and applications of nonlinear registration-based segmentation. Int J Pattern Recognit Artif Intell.

[52] Mawlawi O, Martinez D, Slifstein M, Broft A, Chatterjee R, Hwang DR (2001). Imaging human mesolimbic dopamine transmission with positron emission tomography: I. Accuracy and precision of D(2) receptor parameter measurements in ventral striatum. J Cereb Blood Flow Metab.

[53] Smart K, Cox SML, Kostikov A, Shalai A, Scala SG, Tippler M (2019). Effect of (Z)-isomer content on [11C]ABP688 binding potential in humans. Eur J Nucl Med Mol Imaging.

[54] Smart K, Cox SML, Scala SG, Tippler M, Jaworska N, Boivin M (2019). Sex differences in [11C]ABP688 binding: a positron emission tomography study of mGlu5 receptors. Eur J Nucl Med Mol Imaging.

[55] Haber SN, Knutson B (2010). The reward circuit: linking primate anatomy and human imaging. Neuropsychopharmacology..

[56] Schumann G, Johann M, Frank J, Preuss U, Dahmen N, Laucht M (2008). Systematic analysis of glutamatergic neurotransmission genes in alcohol dependence and adolescent risky drinking behavior. Arch Gen Psychiatry.

[57] Akkus F, Treyer V, Johayem A, Ametamey SM, Mancilla BG, Sovago J (2016). Association of long-term nicotine abstinence with normal metabotropic glutamate receptor-5 binding. Biol Psychiatry..

[58] Minuzzi L, Diksic M, Gauthier S, Quirion R, Rosa-Neto P. In vitro quantification of mGluR5 in pons and cerebellum of human brain using [3H]ABP688. J Cereb Blood Flow Metab. 2009;29:S360–75.

[59] Scala S, Smart K, Cox SML, Leyton M. PET Imaging of metabotropic glutamate receptors. In: Olive F, editor Metabotropic glutamate receptor technologies. Springer; 2020, in press.

[60] Smart K, Cox SML, Nagano-Saito A, Rosa-Neto P, Leyton M, Benkelfat C (2018). Test-retest variability of [11C]ABP688 estimates of metabotropic glutamate receptor subtype 5 availability in humans. Synapse..

[61] Kraemer HC (2015). A source of false findings in published research studies: adjusting for covariates. JAMA Psychiatry.

