# Supplementary material for: mGlu5 receptor availability in youth at risk for addictions: effects of vulnerability traits and cannabis use
Source: Neuropsychopharmacology. 2020 May 15;45(11):1817–25. doi: 10.1038/s41386-020-0708-x (PMC7608187; doi:10.1038/s41386-020-0708-x)
Supplement: Supplementary file 1 — Supplemental material [file 41386_2020_708_MOESM1_ESM.docx]

**Supplemental material**

Table S1 summarizes participant characteristics for the six subgroups. Univariate risk x cannabis use group ANOVAs were conducted to compare externalizing traits, AUDIT scores, cannabis use and other drug use among the groups. Multivariate ANOVAs (followed by univariate ANOVAs) were used to assess differences in externalizing related features between risk and cannabis use groups as measured with the BIS-II, SURPS and SPSRQ.

*Externalizing traits*

As expected, a main effect of risk group was observed on externalizing traits, with higher externalizing traits in the high compared to the low risk group (F(1,53)=188, p<0.001). There was no main effect of cannabis group or a risk x cannabis group interaction (p>0.05).

*Externalizing-related personality traits*

Using MANOVA we observed a significant main effect of risk group on SURPS (Wilk's Lambda: F(4,48)=3.13, p=0.023) and SPSRQ scores (Wilk's Lambda: F(2,50)=3.36, p=0.043) and a strong trend for BIS scores (Wilk's Lambda: F(3,49)=3.66, p=0.059). There were no main effects of cannabis use group or a risk x cannabis group interactions (p>0.05). Follow-up analyses identified significantly higher impulsivity scores as measured by the SURPS (F(1,51)=8.84, p=0.004) and BIS (all subscales: p<0.05) and higher SPSRQ measured sensitivity to punishment (F(1,51)=4.2, p=0.045) and sensitivity to reward scores (F(1,51)=3.8, p=0.056) in the high vs. low risk individuals.

*Cannabis use occasions*

When analyzing cannabis use occasions, the ANOVA yielded the expected main effect of cannabis group (F(2,53)=23, p<0.001) with higher cannabis use in the high cannabis group compared to the low (p<0.001) and zero cannabis (p<0.001) group. A main effect of externalizing risk group approached significance (F(1,53)=4, p=0.052) reflecting higher cannabis use in the high risk compared to the low risk group.

*AUDIT*

A main effect of cannabis group was observed when testing for differences in AUDIT (F(2,53)=6, p=0.01), reflecting higher levels of alcohol use and alcohol related problems in the low (p=0.002) and high (p<0.001) cannabis users compared to those who never used cannabis.

*Drug use other than cannabis*

No significant main or interaction effects were observed for drug use other than cannabis.

Supplementary Table 1: Group characteristics

| Characteristic | Low Risk (n=31) | | | | | | | | | High Risk (n=28) | | | | | | | | |
| --- | --- | --- | --- | --- | --- | --- | --- | --- | --- | --- | --- | --- | --- | --- | --- | --- | --- | --- |
|  | **Zero**  **(n=12)** | | | **Low**  **(n=17)** | | | **High**  **(n=2)** | | | **Zero**  **(n =6)** | | | **Low**  **(n=13)** | | | **High**  **(n=9)** | | |
|  | N | M | SD | N | M | SD | N | M | SD | N | M | SD | N | M | SD | N | M | SD |
| **Age** |  | 18.4 | 0.7 |  | 18.4 | 0.5 |  | 19 | 0 |  | 18.3 | 0.5 |  | 18.4 | 0.7 |  | 19.1 | 0.3 |
| **Sex** | 8f |  |  | 11f |  |  | 1f |  |  | 4f |  |  | 8f |  |  | 4f |  |  |
| **Externalizing traits** |  | 0.36 | 0.3 |  | 0.52 | 0.3 |  | 0.79 | 0.1 |  | 2.43 | 0.4 |  | 2.14 | 0.4 |  | 2.74 | 0.73 |
| **SURPS**  Impulsivity  Hopelessness  Anxiety sensitivity  Sensation seeking | 12  12  12  12 | 7.8  11.1  9  16.7 | 2.7  2.4  3.0  4.6 | 17  17  17  17 | 9.8  11.4  9.5  15.6 | 2.4  3.1  3.2  4.1 | 2  2  2  2 | 9.0  13  12.5  20.5 | 0  1.4  2.1  0.7 | 6  6  6  6 | 11.7  10.1  10.7  15.2 | 2.2  2.6  3.0  2.7 | 12  12  12  12 | 11.1  13  10.1  16.2 | 3.3  3.9  2.3  3.9 | 8  8  8  8 | 11.9  13.8  10.1  18.9 | 2.8  4.5  1.7  3.1 |
| **BIS**  Attention  Motor  Non-planning  Total | 12  12  12  12 | 14.1  17.9  20.3  52.3 | 3.7  3.2  4.6  9.7 | 17  17  17  17 | 14.2  18.2  22.9  55.4 | 3.4  3.1  3.6  6.4 | 2  2  2  2 | 14  21  24.5  59.5 | 0  1.4  3.5  2.1 | 6  6  6  6 | 16.3  22.5  25.2  64 | 2.2  2.2  4.8  7.0 | 12  12  12  12 | 16.1  21.8  24.1  62 | 3.2  4.8  3.3  8.3 | 8  8  8  8 | 16.9  20.6  26.9  64.4 | 3.4  4.0  3.9  9 |
| **SPSRQ**  Reward sensitivity  Punishment sensitivity | 12  12 | 8.3  10.5 | 3.4  4.2 | 17  17 | 8.8  9.2 | 2.8  5.2 | 1  1 | 6  4 | 0  0 | 6  6 | 8.3  13.3 | 2.7  3.9 | 13  13 | 10.5  11.5 | 4.6  4.8 | 8  8 | 13.1  11.5 | 4.6  6.3 |
| **AUDIT** | 12 | 1.9 | 1.6 | 17 | 5.1 | 2.3 | 2 | 7.5 | 2.1 | 6 | 3.7 | 1.6 | 13 | 6.2 | 4.5 | 9 | 7.0 | 4.6 |
| **Cigarette smokers** | 0 |  |  | 1^a^ |  |  | 1^a^ |  |  | 0 |  |  | 0 |  |  | 3^b^ |  |  |
| **Cannabis, lifetime occasions used** |  | 0 |  |  | 8 | 8 |  | 443 | 494 |  | 0 |  |  | 11 | 12.4 |  | 911 | 586 |
| **Cannabis, age of onset** |  |  |  |  | 16.4 | 1.2 |  | 15 | 1.4 |  |  |  |  | 15.7 | 1.4 |  | 15.1 | 1.8 |
| **Cannabis Use within the past month** | 0 |  |  | 0 |  |  | 0 |  |  | 0 |  |  | 1 |  |  | 7 |  |  |
| **Positive THC screen** | 0 |  |  | 0 |  |  | 0 |  |  | 0 |  |  | 0 |  |  | 5 |  |  |
| **Drug Use (excluding cannabis), lifetime occasions** | 0 |  |  | 3 | 3 | 3.5 | 1 | 24 |  | 0 |  |  | 2 | 14 | 17 | 9 | 78 | 144 |
| **Current SUD** | 0 |  |  | 0 |  |  | 0 |  |  | 0 |  |  | 2, mild AUD | | | 4, cannabis, n=3; amphetamine, n=1. | | |
| **Current DSM-5 disorder other than SUD** | 0 |  |  | 0 |  |  | 0 |  |  | 1, dyslexia | | | 1, persistent depressive disorder | | | 3, ADHD, n=1; dyslexia, n=1; ADHD, panic disorder, n=1. | | |
| **Current or past DSM 5 disorder** | 0 |  |  | 1, past MDD | | | 0 |  |  | 1, dyslexia, past ADHD | | | 6, past MDD, n=1; past ADHD, n=1; past adjustment disorder with depressed mood, n=1; current mild AUD and mild past binge eating disorder, n=1; current mild AUD, n=1; current persistent depressive disorder, n=1. | | | 7, past MDD, past adjustment disorder, past panic disorder, n=1; current mild cannabis use disorder, n=1; past and current ADHD, n=1; dyslexia, past ADHD, n=1; past moderate cannabis use disorder, current moderate amphetamine use disorder, n=1; past MDD, AUD, ADHD, current moderate cannabis use disorder, current panic disorder, current ADHD, n=1; past conduct disorder, current moderate cannabis use disorder, n=1. | | |

Zero, Low, and High refer to the cannabis use categories.

^a^ n=1 regular smoker (FTND >2)

^b^ n=1 regular smoker, n=2 occasional social smokers (FTND = 0)

SURPS: Substance Use Risk Profile Scale; BIS: Barratt Impulsiveness Scale; SPSRQ: Sensitivity to Punishment and Sensitivity to Reward Questionnaire; AUDIT: Alcohol Use Disorder Identification Test; THC: delta-9-tetrahydrocannabinol; SUD: Substance Use Disorder; FTND: Fagerström Test for Nicotine Dependence; ADHD: Attention Deficit Hyperactivity Disorder; AUD: Alcohol Use Disorder; MDD: Major Depressive Disorder

Supplementary Table 2: [^11^C]ABP688 BP_ND_ values in cortical and subcortical limbic regions in high and low risk individuals with zero, low and high frequencies of lifetime cannabis use occasions.

| ROI | Low Risk (n=31) | | | | | | High Risk (n=28) | | | | | | Main effect Risk Group^a^ | | Risk x Cannabis Interaction^a^ | |
| --- | --- | --- | --- | --- | --- | --- | --- | --- | --- | --- | --- | --- | --- | --- | --- | --- |
|  | Zero  (n=12) | | Low  (n=17) | | High  (n=2) | | Zero  (n=6) | | Low  (n=13) | | High  (n=9) | | p | Partial η^2^ | P | Partial η^2^ |
|  | Mean* | SD | Mean* | SD | Mean* | SD | Mean* | SD | Mean* | SD | Mean* | SD |  |  |  |  |
| VS | 1.20 | 0.26 | 1.24 | 0.24 | 1.54 | 0.24 | 1.08 | 0.26 | 1.26 | 0.25 | 1.00 | 0.29 | 0.011 | 0.127 | 0.029 | 0.137 |
| AST | 1.15 | 0.24 | 1.14 | 0.22 | 1.48 | 0.22 | 1.03 | 0.24 | 1.20 | 0.23 | 0.95 | 0.26 | 0.013 | 0.122 | 0.009 | 0.177 |
| SMST | 0.93 | 0.19 | 0.90 | 0.17 | 1.12 | 0.18 | 0.88 | 0.19 | 0.93 | 0.18 | 0.71 | 0.20 | 0.023 | 0.103 | 0.018 | 0.154 |
| mOFC | 1.01 | 0.26 | 1.03 | 0.24 | 1.28 | 0.24 | 0.92 | 0.26 | 1.07 | 0.25 | 0.83 | 0.28 | 0.045 | 0.081 | 0.059 | 0.112 |
| lOFC | 0.83 | 0.20 | 0.84 | 0.19 | 1.05 | 0.19 | 0.77 | 0.20 | 0.88 | 0.19 | 0.67 | 0.22 | 0.039 | 0.085 | 0.034 | 0.132 |
| mPFC | 0.86 | 0.24 | 0.93 | 0.23 | 0.95 | 0.23 | 0.86 | 0.24 | 1.0 | 0.23 | 0.81 | 0.27 | 0.109 | 0.002 | 0.549 | 0.025 |
| Amygdala | 0.84 | 0.21 | 0.88 | 0.20 | 1.05 | 0.20 | 0.76 | 0.21 | 0.89 | 0.21 | 0.66 | 0.23 | 0.027 | 0.098 | 0.068 | 0.106 |
| Insula | 1.02 | 0.21 | 1.02 | 0.20 | 1.22 | 0.20 | 0.95 | 0.21 | 1.04 | 0.21 | 0.84 | 0.23 | 0.035 | 0.089 | 0.070 | 0.105 |
| HIPP | 0.63 | 0.19 | 0.71 | 0.17 | 0.88 | 0.17 | 0.62 | 0.18 | 0.75 | 0.18 | 0.59 | 0.20 | 0.073 | 0.065 | 0.085 | 0.098 |

Zero, Low, and High refer to the cannabis use categories. VS= ventral striatum, AST= associative striatum, SMST= somatosensory striatum, mOFC=medial orbitofrontal cortex, lOFC= lateral orbitofrontal cortex, mPFC= medial prefrontal cortex, HIPP = hippocampus.

* Marginal means corrected for variations in sex, isomer ratio, AUDIT scores, tobacco smoking status and drug use other than cannabis.

^a^Results from univariate ANCOVA with risk group and cannabis use group as between subject factors and sex, isomer ratio, AUDIT scores, smoking status and drug use other than cannabis as covariates.
